# Development and validation of a clinical-pharmacogenomic prediction model for clopidogrel-related high on-treatment platelet reactivity in patients with coronary artery disease

**DOI:** 10.3389/fcvm.2026.1926514

**Published:** 2026-09-03

**Authors:** Ruihua Sun, Yu Du

**Affiliations:** Third Department of Coronary Heart Disease, Qinghai Province Cardio Cerebrovascular Disease Specialist Hospital, Xining, Qinghai, China

**Keywords:** antiplatelet therapy, clopidogrel, coronary artery disease, platelet reactivity, predictive model

## Abstract

**Objective:**

This study aimed to develop and validate a prediction model combining clinical indicators and pharmacogenomic markers for predicting clopidogrel-related high on-treatment platelet reactivity (HPR) after approximately 7 days of dual antiplatelet therapy in patients with coronary artery disease (CAD), and to identify its influencing factors to support individualized antiplatelet therapy. The model is intended for use before platelet function testing results are available, to identify patients at high risk of HPR who may benefit from early testing or genotype-guided therapy adjustment.

**Methods:**

A total of 380 CAD patients were retrospectively enrolled and divided into a training set (*n* = 266) and a validation set (*n* = 114) at a 7:3 ratio. HPR was defined as ADP-induced platelet aggregation ≥40%. We screened predictive variables via univariate analysis and LASSO regression, then identified independent factors using multivariate logistic regression. Four prediction models were established and compared. Model performance was assessed by area under the receiver operating characteristic curve (AUC), calibration curve, decision curve analysis and SHAP values.

**Results:**

Seven core predictive variables were identified. Multivariate logistic regression analysis revealed that *CYP2C19* functional deletion allele carrier status (OR = 4.341, *P* < 0.001), PPI use (OR = 2.630,*P* = 0.007), acute coronary syndrome (OR = 2.445,*P* = 0.014), age (OR = 1.053, *P* = 0.006), and platelet count (OR = 1.014, *P* < 0.001), left ventricular ejection fraction (OR = 0.929, *P* = 0.001) and hemoglobin (OR = 0.952, *P* < 0.001) were independent factors. Among the four prediction models, Logistic regression model demonstrated optimal performance, with an AUC value of 0.866 (95% CI: 0.816-0.916) on the training set and 0.870(95% CI:0.793-0.946) on the validation set. The model showed good calibration and clinical net benefit, and *CYP2C19* genotype exerted the greatest predictive impact.

**Conclusion:**

This study developed and internally validated a clinical-pharmacogenomic prediction model for clopidogrel-related HPR in CAD patients. The model demonstrated promising performance; however, external validation in multicenter prospective cohorts is required before clinical implementation.

## 1 Introduction

Coronary artery disease (CAD) represents a major cardiovascular condition that poses a serious threat to human health. Following percutaneous coronary intervention (PCI), long-term dual antiplatelet therapy (DAPT) is required to prevent thrombotic events (1). Currently, the combination of aspirin and a P2Y12 receptor inhibitor constitutes the standard antithrombotic regimen for patients with acute coronary syndrome (ACS) and those undergoing PCI. However, clinical practice has revealed that a subset of patients experience thrombotic events despite receiving standard-dose antiplatelet therapy, a phenomenon termed high on-treatment platelet reactivity (HPR). Studies indicate that approximately 20% to 40% of patients exhibit HPR, which is associated with a significantly increased risk of cardiovascular death, myocardial infarction, and stent thrombosis. The mechanisms underlying HPR are complex and involve multiple processes, including drug metabolism, absorption, and activation (2). P2Y12 receptor inhibitors require metabolic conversion by the hepatic cytochrome P450 enzyme system (primarily *CYP2C19*) to generate active metabolites. Consequently, *CYP2C19* polymorphisms represent a key genetic determinant of antiplatelet efficacy (3). Carriers of loss-of-function *CYP2C19* alleles exhibit reduced drug metabolism, diminished generation of active metabolites, impaired platelet inhibition, and a significantly elevated risk of thrombotic events (4). In addition to *CYP2C19*, other genetic variants have been investigated for their potential roles in clopidogrel response. *ABCB1* encodes *P*-glycoprotein, which affects the intestinal absorption of clopidogrel, and *PON1* encodes paraoxonase-1, which has been suggested to participate in clopidogrel bioactivation. Although the evidence for these variants has been inconsistent across studies, we included them as exploratory pharmacogenomic markers.Beyond genetic factors, clinical characteristics also play a crucial role in the response to antiplatelet therapy. Advanced age, diabetes mellitus, acute coronary syndrome, and a heightened inflammatory state can each contribute to increased platelet reactivity (5). Furthermore, proton pump inhibitors (particularly omeprazole and esomeprazole) may reduce the efficacy of certain antiplatelet agents by competing for the *CYP2C19* metabolic enzyme, thereby further increasing the risk of HPR.

Although existing prediction tools such as the ABCD-GENE score and GeneFA score have been developed for clopidogrel response prediction, they primarily focus on genetic factors and limited clinical variables. There remains a need for models that integrate a broader range of clinical indicators—including LVEF, hemoglobin, and platelet count—with pharmacogenomic markers to provide more comprehensive individualized risk assessment at the early stage of antiplatelet therapy，before platelet function testing results are available. Least absolute shrinkage and selection operator (LASSO) regression, an effective feature selection method, can identify the most predictive variables from high-dimensional data while mitigating model overfitting.Based on this, this study retrospectively enrolled 380 patients with CAD, collecting their baseline data, clinical characteristics, laboratory parameters, and pharmacogenomic data. LASSO regression was employed to screen core predictive variables for hyperplatelet reactivity. On this basis, four prediction models—random forest, support vector machine, Logistic regression model and gradient-boosting tree —were constructed and compared. The models' discriminative power, calibration, and clinical net benefit were evaluated through internal validation. The aim was to provide objective evidence for clinicians to identify high-risk populations early and develop individualized antiplatelet treatment regimens.

## 2 Methods

### 2.1 Study population

This investigation was a single-center, retrospective cohort study conducted at Qinghai Province Cardio Cerebrovascular Disease Specialist Hospital. A total of 486 patients diagnosed with CAD who were admitted to the Department of Cardiology between January 2022 and June 2024 were initially screened. After excluding 50 patients who did not meet the clinical inclusion criteria (18 with severe liver or kidney disease, 22 with recent use of other antiplatelet or anticoagulant agents, 8 with active bleeding or bleeding disorders, and 2 who were pregnant or lactating), 436 patients remained. Of these, 24 patients did not undergo platelet function testing, leaving 412 patients with platelet function results. Subsequently, 14 patients did not undergo pharmacogenomic testing, yielding 398 patients with both platelet function and genotyping data. Finally, 18 patients were excluded due to incomplete clinical data, resulting in 380 patients for final analysis. These 380 patients were randomly assigned to the training set (*n* = 266) and validation set (*n* = 114) in a 7:3 ratio. The participant flow diagram is presented in Supplementary Figure 1. In our center, CYP2C19 genotyping and platelet function testing have been routinely performed for all CAD patients prescribed clopidogrel-based DAPT since 2021 as part of an institutional quality improvement initiative; therefore, testing was not initiated because of suspected clopidogrel nonresponse. All patients were of Han Chinese ethnicity, as documented in their medical records.This study was approved by the institutional ethics review committee. Given the retrospective nature of the study and the anonymization of all data, the requirement for patient informed consent was waived following review by the ethics committee.

Based on sample size estimation principles for prediction model studies, a minimum of 10 to 15 outcome events per predictor variable is required. This study included 7 predictor variables, with 116 patients in the HPR group, yielding an events per variable (EPV) of 16.6, which satisfied the requirements for model development.

Inclusion criteria were: ① age ≥18 years; ② confirmed diagnosis of CAD (including stable CAD and ACS); ③ receipt of clopidogrel-based DAPT (clopidogrel plus aspirin) for at least 7 days: ACS patients received a loading dose of clopidogrel 300 mg followed by maintenance therapy of clopidogrel 75 mg daily plus aspirin 100 mg daily; stable CAD patients received maintenance therapy of clopidogrel 75 mg daily plus aspirin 100 mg daily from admission. Platelet function testing was performed after at least 7 days of maintenance therapy. Medication adherence was confirmed through electronic prescription records and nursing documentation; ④ completed platelet function testing (adenosine diphosphate-induced platelet aggregation); and ⑤ complete clinical data, including baseline information, laboratory parameters, and pharmacogenomic data.

Exclusion criteria were: ① concomitant severe liver disease (alanine aminotransferase ≥3 times the upper limit of normal) or severe kidney disease (estimated glomerular filtration rate <30 mL/min/1.73 m²); ② recent use of other antiplatelet or anticoagulant medications affecting platelet function assessment; ③ presence of active bleeding or bleeding disorders; ④ pregnancy or lactation; and ⑤ incomplete clinical data precluding the determination of platelet reactivity status.

### 2.2 Data collection

Systematic collection of multi-dimensional data was performed using our hospital's Picture Archiving and Communication System, electronic medical record system, and laboratory database to construct the pharmacogenomic prediction model for antiplatelet therapy response. All clinical characteristics and laboratory parameters were measured at baseline before clopidogrel initiation. *CYP2C19* genotype, as a germline genetic variant, was assessed at any time point during hospitalization. Platelet function testing (ADP-induced platelet aggregation) was performed after at least 7 days of continuous clopidogrel-based DAPT, with blood samples collected in the fasting state in the morning and testing completed within 2 h of collection. This temporal sequence ensures that all predictor variables precede the outcome assessment. Data collection encompassed the following aspects: (1) Clinical Baseline Data: Demographic characteristics, including age, sex, and body mass index; lifestyle information, including smoking history and alcohol consumption history; and clinical features, including CAD type (stable CAD/ACS), history of hypertension, history of diabetes mellitus, history of renal insufficiency, history of prior PCI or coronary artery bypass grafting, and left ventricular ejection fraction were extracted from the electronic medical record system. (2) Laboratory Parameters: Pre-treatment laboratory results, including platelet count, hemoglobin, liver function (alanine aminotransferase), lipid profile (low-density lipoprotein cholesterol), and C-reactive protein levels were collected from the laboratory database. All laboratory parameters were measured using standardized methods in our hospital's clinical laboratory. (3) Pharmacogenomic Data: Results of *CYP2C19*, *ABCB1*, and *PON1* polymorphism testing were collected. All genotyping was uniformly performed using the improved multiplex ligation detection reaction (iMLDR) method, strictly following the manufacturer’s instructions. Results were independently interpreted by two experienced laboratory physicians. (4) Medication Information and Platelet Function Testing: Information on proton pump inhibitor (PPI) use was extracted from the prescription order system. The specific PPIs prescribed included omeprazole (*n* = 52, 45.6%), esomeprazole (*n* = 38, 33.3%), pantoprazole (*n* = 16, 14.0%), and rabeprazole (*n* = 8, 7.0%). Due to the retrospective design and limited sample size for each subtype, PPI use was coded as a binary variable in the primary analysis. Adenosine diphosphate-induced platelet aggregation values were obtained from platelet function test reports, with HPR defined as platelet aggregation ≥40%. All clinical data and laboratory parameters were independently entered into an Excel database by two researchers and cross-verified to ensure data accuracy. Discrepancies were resolved through discussion with a third researcher, providing a reliable foundation for subsequent feature selection and model development. Genomic DNA was extracted from peripheral blood samples using a commercial DNA extraction kit (Tiangen Biotech, Beijing, China). All genotyping was uniformly performed using the improved multiplex ligation detection reaction (iMLDR) method (Genesky Biotechnologies Inc., Shanghai, China). The following variants were genotyped: CYP2C19 *2 (rs4244285), CYP2C19 *3 (rs4986893), CYP2C19 *17 (rs12248560), ABCB1 rs1045642, and PON1 rs662. Each run included duplicate samples and negative controls for quality assurance. Genotype call rates exceeded 98% for all variants (CYP2C19 *2: 99.2%; CYP2C19 *3: 98.9%; CYP2C19 *17: 98.7%; ABCB1 rs1045642: 99.0%; PON1 rs662: 98.5%). Hardy-Weinberg equilibrium was assessed for all variants using the *χ*² test among all included patients; all variants were in HWE (all *P* > 0.05). No missing genotyping data were present for the included patients.

Based on the combination of CYP2C19 alleles, patients were classified according to CPIC guidelines: *1/*1 as normal metabolizers, *1/*2 or *1/*3 as intermediate metabolizers, and *2/*2, *2/*3, or *3/*3 as poor metabolizers. For the primary analysis, carriers of at least one loss-of-function allele (*2 or *3) were classified as the ‘carrier’ group. In a supplementary analysis, we evaluated the dose-response relationship by stratifying patients according to the number of loss-of-function alleles (0, 1, or 2 alleles).

### 2.3 Definitions of clinical variables

The following operational definitions were used: Acute coronary syndrome (ACS) was defined as ST-segment elevation myocardial infarction, non-ST-segment elevation myocardial infarction, or unstable angina. Stable coronary artery disease was defined as documented coronary stenosis ≥50% in at least one major epicardial vessel without acute ischemic symptoms within the preceding 2 months. Hypertension was defined as systolic blood pressure ≥140 mmHg and/or diastolic blood pressure ≥90 mmHg on at least two separate occasions, or current use of antihypertensive medication. Diabetes mellitus was defined as fasting plasma glucose ≥7.0 mmol/L, or 2-hour plasma glucose ≥11.1 mmol/L during oral glucose tolerance test, or current use of glucose-lowering medication. Renal insufficiency was defined as estimated glomerular filtration rate <60 mL/min/1.73 m². Smoking history was defined as current smoking or cessation within the past year. Alcohol consumption was defined as alcohol intake ≥1 time per week for at least 6 months. Prior PCI or CABG was confirmed by review of procedural records. Elevated C-reactive protein was defined as ≥3 mg/L. Left ventricular ejection fraction was measured using the biplane Simpson method on echocardiography performed within 48 h of admission. All laboratory parameters (platelet count, hemoglobin, ALT, LDL-C, CRP, and renal function) were measured at baseline before clopidogrel initiation. Platelet function testing was performed after at least 7 days of continuous clopidogrel-based DAPT.

### 2.4 Outcome definition

This study strictly adhered to platelet function testing standards, with the endpoint outcome clearly defined based on platelet aggregation results. All cases were categorized according to the following detailed criteria: 1. Diagnostic Criteria for the High Platelet Reactivity (HPR) Group (Case Group): HPR was defined as inadequate platelet inhibition following antiplatelet therapy, requiring fulfillment of the following conditions: (1) Platelet function testing criteria: HPR was defined as maximal platelet aggregation ≥40% using light transmission aggregometry (LTA) with 5 μmol/L adenosine diphosphate (ADP) as the agonist (Chrono-log 700 aggregometer, Chrono-log Corporation, Havertown, PA, USA), with maximal aggregation recorded within 5 min after ADP stimulation. This cutoff was prespecified based on previous studies specifically conducted in Chinese CAD populations, which identified 40% as the optimal threshold for predicting ischemic events (6). All platelet function tests were performed in a fasting state in the morning, with testing completed within 2 h of blood collection to ensure accuracy and reliability. (2) Confirmation of medication adherence: Patients were confirmed to be taking antiplatelet medications regularly, with good adherence and no missed or discontinued doses. Medication information was verified through dual-source confirmation using the electronic prescription system and nursing records. (3) Exclusion of confounding factors: No use of other medications affecting platelet function, no active bleeding events, and no history of blood transfusion within one week prior to testing. 2. Diagnostic Criteria for the Normal Platelet Reactivity Group (Control Group): Normal platelet reactivity was defined as adequate platelet inhibition following antiplatelet therapy, requiring fulfillment of the following conditions: (1) Platelet function testing criteria: Platelet aggregation rate <40%. (2) Confirmation of medication adherence: Same as the case group, patients were confirmed to be taking antiplatelet medications regularly with good adherence. (3) Exclusion of confounding factors: Same as the case group, no use of medications affecting platelet function, no active bleeding events, and no history of blood transfusion within one week prior to testing. 3. Exclusion Criteria for Group Assignment: The following cases were not included in the group analysis: ① Unclear or incomplete platelet aggregation test results; ② History of blood transfusion within one week prior to testing; ③ Concomitant hematologic disorders affecting platelet count or function; ④ Concurrent use of other medications affecting platelet function; ⑤ Severe hepatic or renal insufficiency potentially affecting drug metabolism; and ⑥ Incomplete clinical data precluding confirmation of medication adherence. Case screening and grouping were independently performed by two cardiologists blinded to the study hypothesis, based on complete clinical data, medication records, and platelet function test reports. Disagreements were resolved by arbitration from a third senior attending physician. All grouping information was double-checked to ensure the accuracy and reliability of outcome labels used for model development and validation.

### 2.5 Statistical analysis

Data analysis was performed using SPSS version 26.0, R software (version 4.2.3), and Python (version 3.8.5). Continuous variables following a normal distribution were presented as mean ± standard deviation (x¯ ± s), and comparisons between two groups were conducted using the independent samples t-test. Categorical variables were presented as numbers (percentages) [n (%)], and comparisons between two groups were performed using the χ² test. All enrolled CAD patients were randomly divided into a training set (*n* = 266) and a validation set (*n* = 114) in a 7:3 ratio using stratified sampling to preserve the distribution of the outcome variable (HPR vs. NPR) in both sets. The 70/30 split was chosen to provide an adequate sample size for model training while retaining a sufficiently large independent sample for internal validation. In the training set, univariate analysis was initially performed to identify variables associated with HPR (*P* < 0.05). Variables achieving statistical significance were then incorporated into multivariate logistic regression analysis to determine independent factors associated with HPR, calculating their regression coefficients (B), odds ratios (ORs), and 95% confidence intervals (CIs). Sample size calculation adhered to prediction model guidelines, requiring at least 10 to 15 events per variable (EPV). With 116 cases in the HPR group and a planned inclusion of no more than 10 variables, the EPV exceeded 10, satisfying model development requirements. Multicollinearity among variables was assessed using the variance inflation factor (VIF), with all variables demonstrating VIF <2, indicating no significant multicollinearity. Based on core variables screened through LASSO regression, multivariate logistic regression analysis was employed to identify independent influencing factors of hyperplatelet reactivity, with regression coefficients (B), odds ratios (OR), and 95% confidence intervals (CI) calculated. Building upon this foundation, four predictive models—random forest, support vector machine, Logistic regression model，and gradient boosting tree—were constructed using the scikit-learn library in Python. Model hyperparameters were optimized through grid search combined with 5-fold cross-validation. Model discrimination was evaluated using the area under the receiver operating characteristic curve (AUC), where an AUC value closer to 1 indicated better discriminatory ability. Calibration curves were plotted to assess the agreement between predicted probabilities and observed outcomes, with the Hosmer-Lemeshow goodness-of-fit test used to evaluate model calibration (*P* > 0.05 indicating good calibration). Decision curve analysis (DCA) was performed to evaluate the clinical net benefit of the model across different threshold probabilities, quantifying its clinical utility. Based on the multivariate logistic regression results, the complex regression equation was transformed into an intuitive graphical tool to facilitate clinical application. Internal validation was performed using the Bootstrap resampling method (1,000 repetitions), calculating the bias-corrected C-index and calibration curves to assess model stability and reliability.

## 3 Results

### 3.1 Comparison of baseline characteristics between training and validation sets

A total of 380 CAD patients were included in this study and were randomly divided into a training set (*n* = 266) and a validation set (*n* = 114) in a 7:3 ratio. Supplementary Figure 1 presents the participant flow diagram depicting the full screening and exclusion procedure. Within the training set, 116 patients (43.6%) were classified into the HPR group, and 150 patients (56.4%) were classified into the normal platelet reactivity group. The validation set included 50 patients with HPR and 64 patients with NPR, with a total of 114 patients. Comparisons of baseline characteristics between the training and validation sets, including age, sex, body mass index, smoking history, alcohol consumption history, CAD type (stable CAD/ACS), history of hypertension, history of diabetes mellitus, history of renal insufficiency, history of prior PCI/CABG, left ventricular ejection fraction, platelet count, hemoglobin, liver function, lipid levels, elevated C-reactive protein, carriage of *CYP2C19* loss-of-function alleles, *ABCB1* polymorphisms, *PON1* polymorphisms, and proton pump inhibitor use, revealed no statistically significant differences (*P* > 0.05) (Table 1). Hardy-Weinberg equilibrium testing showed that all genotyped variants—CYP2C19 *2 (rs4244285), *3 (rs4986893), *17 (rs12248560), ABCB1 rs1045642, and PON1 rs662—were in Hardy-Weinberg equilibrium (all *P* > 0.05). The genotype and allele frequencies for all genotyped variants are presented in Supplementary Table 1.

**Table 1:** Comparison of baseline characteristics between training and validation sets.

Variable	Training set (*n* = 266)	Validation set (*n* = 114)	t/χ²	*P*
Age (years)	62.53 ± 10.18	61.84 ± 9.77	0.613	0.540
Sex [*n* (%)]			0.029	0.866
Male	182 (68.42)	79 (69.30)		
Female	84 (31.58)	35 (30.70)		
BMI (kg/m²)	25.83 ± 3.22	26.14 ± 3.41	0.845	0.399
Smoking history [*n* (%)]			0.002	0.963
Yes	108 (40.60)	46 (40.35)		
No	158 (59.40)	68 (59.65)		
Alcohol consumption history [*n* (%)]			0.015	0.903
Yes	80 (30.08)	35 (30.70)		
No	186 (69.92)	79 (69.30)		
Type of CAD [*n* (%)]			0.002	0.963
Stable coronary artery disease	108 (40.60)	46 (40.35)		
Acute coronary syndrome	158 (59.40)	68 (59.65)		
History of hypertension [*n* (%)]			0.005	0.945
Yes	160 (60.15)	69 (60.53)		
No	106 (39.85)	45 (39.47)		
History of diabetes mellitus [*n* (%)]			0.002	0.961
Yes	80 (30.08)	34 (29.82)		
No	186 (69.92)	80 (70.18)		
History of renal insufficiency [*n* (%)]			0.001	0.975
Yes	40 (15.04)	17 (14.91)		
No	226 (84.96)	97 (85.09)		
History of prior PCI/CABG [*n* (%)]			0.003	0.959
Yes	67 (25.19)	29 (25.44)		
No	199 (74.81)	85 (74.56)		
LVEF (%)	54.18 ± 8.52	55.03 ± 9.12	0.872	0.384
Platelet count (×10⁹/L)	225.63 ± 48.32	218.92 ± 51.21	1.218	0.224
Hemoglobin (g/L)	136.24 ± 14.53	134.81 ± 15.33	0.865	0.389
Liver function (ALT, U/L)	24.82 ± 9.63	25.43 ± 10.22	0.556	0.579
Lipids (LDL-C, mmol/L)	2.45 ± 0.76	2.53 ± 0.82	0.918	0.359
Elevated C-reactive protein [*n* (%)]			0.008	0.927
Yes (≥3 mg/L)	106 (39.85)	46 (40.35)		
No (<3 mg/L)	160 (60.15)	68 (59.65)		
*CYP2C19* loss-of-function allele carriage [*n* (%)]			0.005	0.947
Carrier	132 (49.62)	57 (50.00)		
Non-carrier	134 (50.38)	57 (50.00)		
*ABCB1* polymorphism [*n* (%)]			0.017	0.992
CC	66 (24.81)	28 (24.56)		
CT	134 (50.38)	57 (50.00)		
TT	66 (24.81)	29 (25.44)		
*PON1* polymorphism [*n* (%)]			0.007	0.997
QQ	130 (48.87)	56 (49.12)		
QR	112 (42.11)	48 (42.11)		
RR	24 (9.02)	10 (8.77)		
PPI use [*n* (%)]			0.002	0.961
Yes	80 (30.08)	34 (29.82)		
No	186 (69.92)	80 (70.18)		

In a supplementary analysis evaluating the dose-response relationship by number of CYP2C19 loss-of-function alleles, the prevalence of HPR was 24.6% (31/126) among non-carriers, 52.4% (55/105) among carriers of one loss-of-function allele, and 73.6% (28/38) among carriers of two loss-of-function alleles (*P* for trend < 0.001).

### 3.2 Univariate analysis of factors influencing high on-treatment platelet reactivity

Among the 266 patients in the training set, 116 (43.6%) were classified into the HPR group, and 150 (56.4%) were classified into the NPR group. In the training set, statistically significant differences were observed between the HPR and NPR groups in terms of age, type of CAD, LVEF, platelet count, hemoglobin level, carriage of *CYP2C19* loss-of-function alleles, and PPI use (*P* < 0.05). Conversely, no statistically significant differences were found in sex, body mass index, history of diabetes mellitus, history of renal insufficiency, liver function, blood lipids, or *ABCB1* gene polymorphisms (*P* > 0.05) **(**Table 2**)**.

**Table 2:** Univariate analysis of factors influencing high on-treatment platelet reactivity.

Variable	Normal platelet reactivity Group (*n* = 150)	High platelet reactivity Group (*n* = 116)	*t/χ²*	*P*
Age (years)	60.28 ± 9.34	65.47 ± 10.82	4.193	0.001
Sex [*n* (%)]			0.490	0.484
Male	100 (66.67)	82 (71.93)		
Female	50 (33.33)	34 (28.07)		
BMI (kg/m²)	25.92 ± 3.18	25.71 ± 3.27	0.528	0.598
Smoking history [*n* (%)]			0.052	0.820
Yes	60 (40.00)	48 (41.38)		
No	90 (60.00)	68 (58.62)		
Alcohol consumption history [*n* (%)]			0.001	0.976
Yes	45 (30.00)	35 (30.17)		
No	105 (70.00)	81 (69.83)		
Type of CAD [*n* (%)]			5.247	0.022
Stable coronary artery disease	70 (46.67)	38 (32.76)		
Acute coronary syndrome	80 (53.33)	78 (67.24)		
History of hypertension [*n* (%)]			0.003	0.956
Yes	90 (60.00)	70 (60.36)		
No	60 (40.00)	46 (39.66)		
History of diabetes mellitus [*n* (%)]			0.606	0.436
Yes	48 (32.00)	32 (27.59)		
No	102 (68.00)	84 (72.41)		
History of renal insufficiency [*n* (%)]			1.513	0.219
Yes	19 (12.67)	21 (18.10)		
No	131 (87.33)	95 (81.90)		
History of prior PCI/CABG [*n* (%)]			0.399	0.528
Yes	40 (26.67)	27 (23.28)		
No	110 (73.33)	89 (76.72)		
LVEF(%)	56.43 ± 7.89	51.56 ± 8.94	4.7094	0.001
Platelet count (×10⁹/L)	213.45 ± 45.67	240.18 ± 50.34	4.526	0.001
Hemoglobin (g/L)	140.82 ± 13.65	131.19 ± 15.42	4.529	0.001
Liver function (ALT, U/L)	25.13 ± 9.42	24.42 ± 9.91	0.596	0.552
Lipids (LDL-C, mmol/L)	2.41 ± 0.73	2.51 ± 0.79	1.069	0.286
Elevated C-reactive protein [*n* (%)]			0.003	0.955
Yes (≥3 mg/L)	60 (40.00)	46 (39.66)		
No (<3 mg/L)	90 (60.00)	70 (60.34)		
*CYP2C19* loss-of-function allele carriage [*n* (%)]			16.520	0.001
Carrier	58 (38.67)	74 (63.79)		
Non-carrier	92 (61.33)	42 (36.21)		
*ABCB1* polymorphism [*n* (%)]			0.403	0.817
CC	38 (25.33)	28 (24.14)		
CT	77 (51.33)	57 (49.14)		
TT	35 (23.34)	31 (26.72)		
*PON1* polymorphism [*n* (%)]			0.010	0.954
QQ	73 (48.66)	57 (49.13)		
QR	63 (42.00)	49 (42.24)		
RR	14 (9.34)	10 (8.62)		
PPI use [n (%)]			16.603	0.001
Yes	30 (20.00)	50 (43.10)		
No	120(80.00)	66(56.90)		

### 3.3 Multivariate logistic regression analysis of factors influencing high on-treatment platelet reactivity

**Figure 1:**
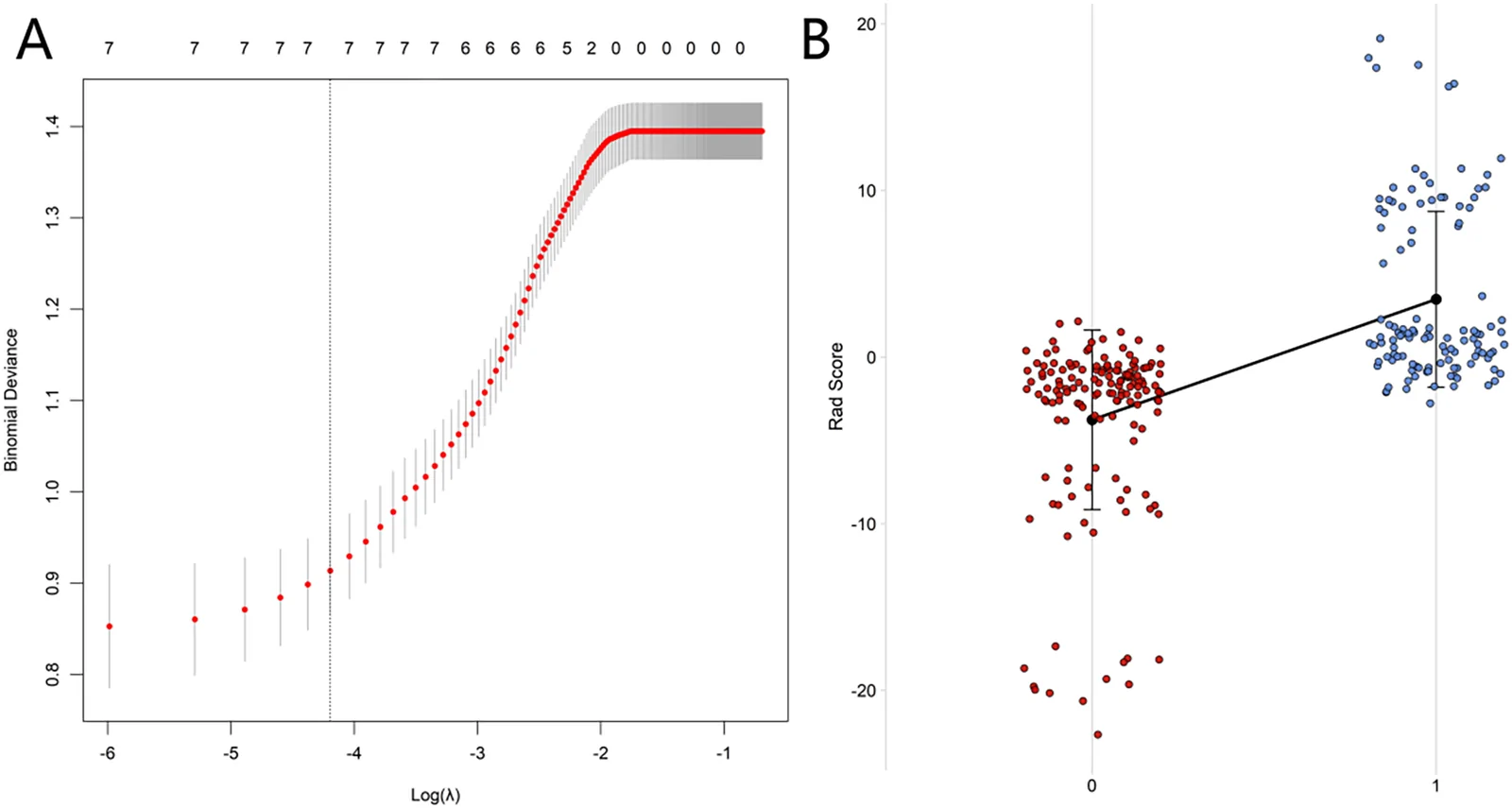
LASSO regression analysis. **(A)** Cross-validation error plot; **(B)** LASSO score plot.

Using high on-treatment platelet reactivity as the dependent variable (1 = HPR group, 0 = NPR group), the seven variables that were statistically significant in the univariate analysis (age, type of CAD, LVEF, platelet count, hemoglobin level, carriage of *CYP2C19* loss-of-function alleles, and PPI use) were all incorporated into a LASSO regression for variable selection (variable assignments are shown in Supplementary Table 2 and Figure 1). Optimal variables were selected using 10-fold cross-validation with the *λ*-1se criterion. Consequently, all seven predictor variables were retained and subsequently included in the multivariate logistic regression model. The results of the multivariate logistic regression analysis (Table 3) indicated that age, type of CAD, LVEF, platelet count, hemoglobin level, carriage of *CYP2C19* loss-of-function alleles, and PPI use were independent influencing factors for HPR (*P* < 0.05). The complete logistic regression prediction equation was: Logit (P) = −4.236 + 0.052 × Age+0.894 × Type of CAD+(−0.074) × LVEF+0.014×Platelet_count+(−0.049)×Hemoglobin+1.468×*CYP2C19*_carrier+0.967×PPI_use, where *P* represents the predicted probability of HPR, Type of CAD is coded as 0 for stable CAD and 1 for ACS, *CYP2C19*_carrier is coded as 0 for non-carrier and 1 for carrier, and PPI use is coded as 0 for no and 1 for yes.

**Table 3:** Multivariate logistic regression analysis of factors influencing high on-treatment platelet reactivity.

Variable	*β*	SE	Wald	*P*	OR	95%CI
Age	0.052	0.019	7.453	0.006	1.053	1.015–1.093
Type of CAD	0.894	0.362	6.098	0.014	2.445	1.202–4.971
LVEF	−0.074	0.023	10.145	0.001	0.929	0.888–0.972
Platelet count	0.014	0.004	13.047	0.001	1.014	1.006–1.021
Hemoglobin	−0.049	0.013	15.343	0.001	0.952	0.928–0.977
Carriage of *CYP2C19* loss-of-function alleles	1.468	0.361	16.520	0.001	4.341	2.138–8.814
PPI use	0.967	0.361	7.179	0.007	2.630	1.296–5.337

### 3.4 Performance evaluation of prediction models

**Figure 2:**
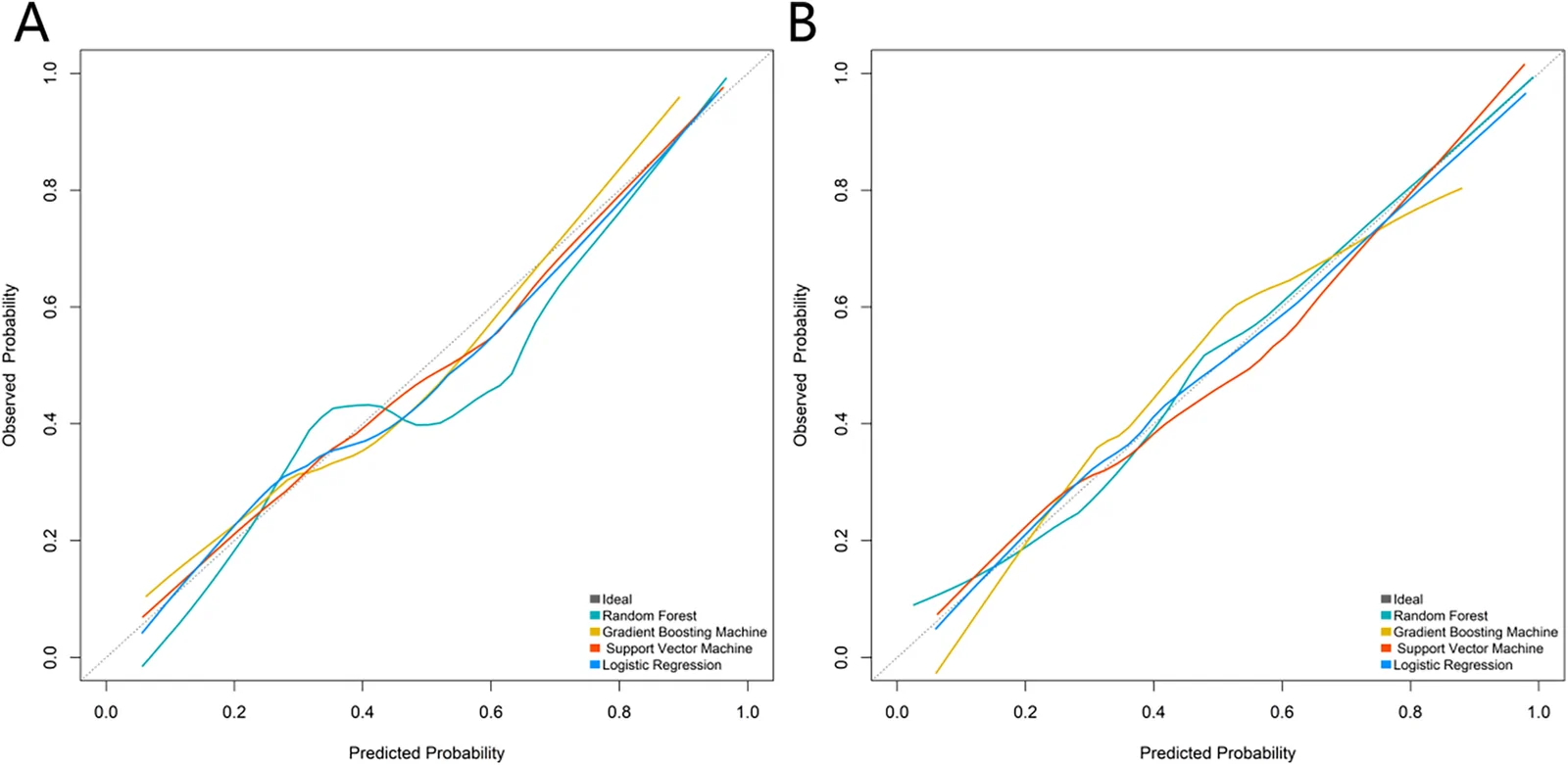
ROC curve analysis of the prediction model in the training **(A)** and validation **(B)** sets.

**Figure 3:**
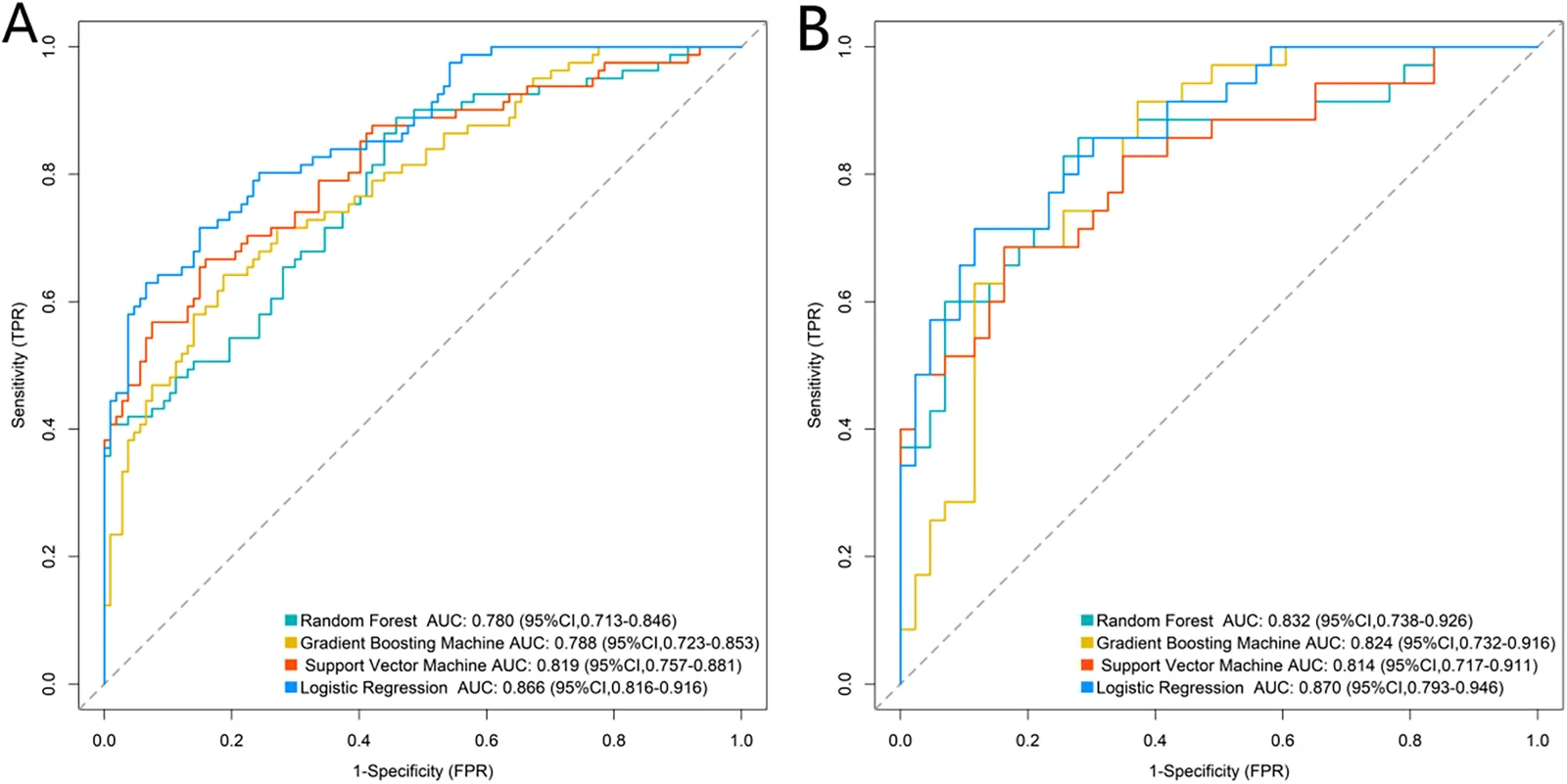
Calibration curve analysis of the prediction model in the training **(A)** and validation **(B)** sets.

**Figure 4:**
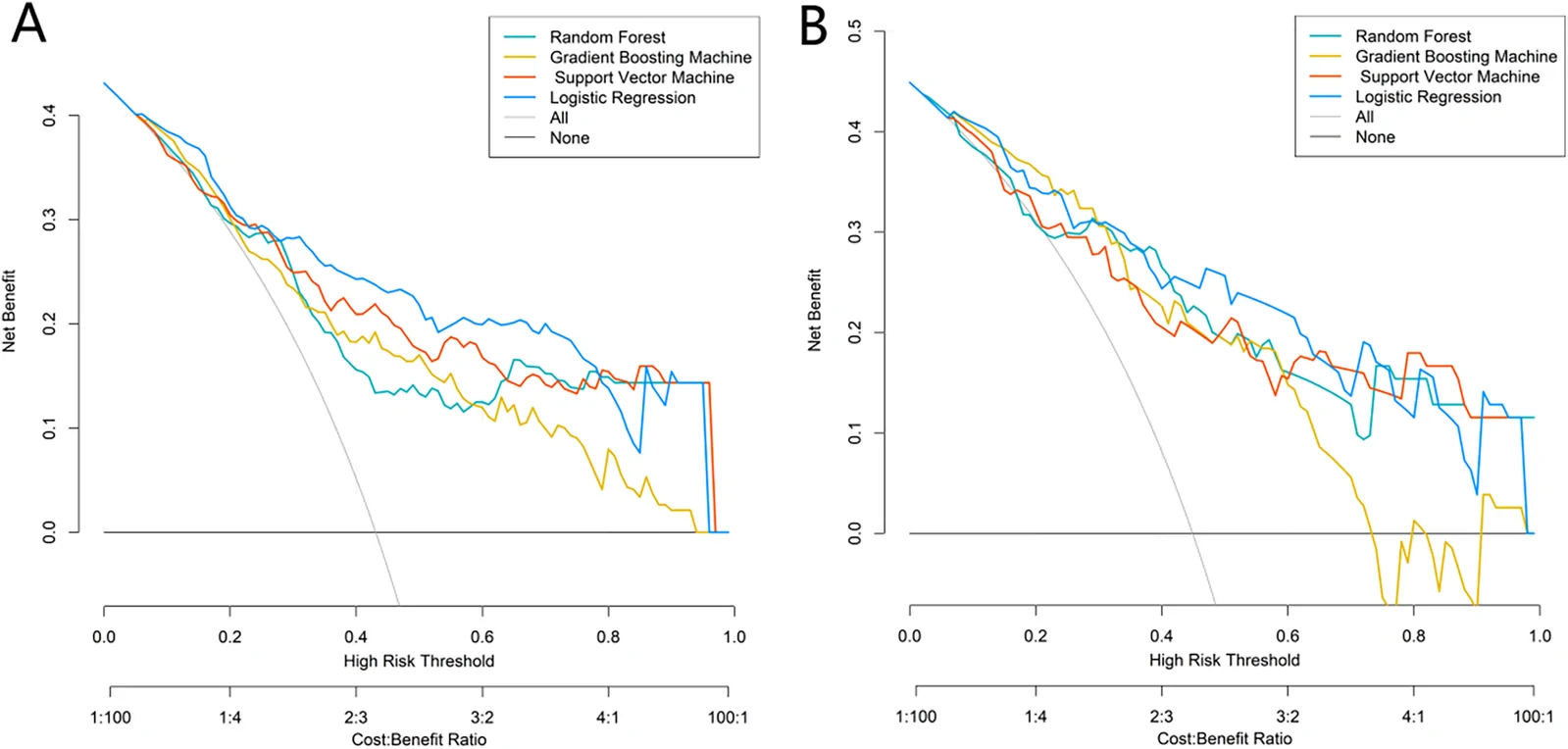
Clinical decision curve analysis of the prediction model in the training **(A)** and validation **(B)** sets.

Based on the seven core predictive factors identified through the preceding univariate and multivariate analyses (age, type of CAD, LVEF, platelet count, hemoglobin level, carriage of *CYP2C19* loss-of-function alleles, and PPI use), this study further developed four prediction models: Random Forest, Support Vector Machine, Logistic regression model，and Gradient Boosting Machine. Their predictive performance was systematically evaluated in both the training and validation sets using Receiver Operating Characteristic (ROC) curves (Figure 2), calibration curves (Figure 3), and decision curves (Figure 4). In the ROC curve analysis, as shown in Figure 2A, Logistic regression model achieved the highest Area Under the Curve (AUC) value of 0.866 (95% CI: 0.816–0.916) in the training set, outperforming Support Vector Machine (0.819) and Gradient Boosting Machine (0.788),and Random Forest (0.780). Similarly, in the validation set (Figure 2B), Logistic regression model maintained the highest AUC of 0.870 (95% CI: 0.793–0.946), demonstrating superior and stable discriminatory ability compared to the Random Forest(0.832), Gradient Boosting Machine (0.824) and Support Vector Machine (0.814). Calibration curve analysis revealed that the predicted probability curve for the Logistic regression model most closely aligned with the ideal diagonal in the training set (Figure 3A). This good fit was sustained in the validation set (Figure 3B), indicating high consistency between predicted risks and observed outcomes, signifying excellent calibration. Calibration metrics further confirmed good model calibration. In the training set, the calibration slope was 0.982 (95% CI: 0.912–1.052), calibration intercept was −0.015 (95% CI: −0.089 to 0.059), and Brier score was 0.176. In the validation set, the calibration slope was 0.965 (95% CI: 0.873–1.057), calibration intercept was 0.008 (95% CI: −0.092 to 0.108), and Brier score was 0.183. Decision curve analysis (Figures 4A,B) demonstrated that the logistic regression model provided positive net benefit across a wide range of clinically relevant threshold probabilities. In the training set, the model showed positive net benefit across threshold probabilities from approximately 0.10 to 0.80, with maximum net benefit of 0.32 achieved at a threshold probability of 0.35. In the validation set, the model showed positive net benefit across threshold probabilities from 0.12 to 0.78, with maximum net benefit of 0.29 at a threshold probability of 0.38. These findings indicate that using the model to guide clinical decisions—such as whether to perform platelet function testing or adjust antiplatelet therapy—would result in greater clinical benefit than the “treat-all” or “treat-none” strategies across most clinically relevant risk thresholds. In summary, Logistic regression model, developed using clinical indicators and pharmacogenomic markers, demonstrates robust discriminative ability, calibration, and clinical net benefit for predicting high on-treatment platelet reactivity in patients with CAD. This model provides clinicians with an objective and reliable quantitative basis for early identification of high-risk individuals and the formulation of individualized antiplatelet therapy strategies.

### 3.5 Interpretability assessment of model predictions

**Figure 5:**
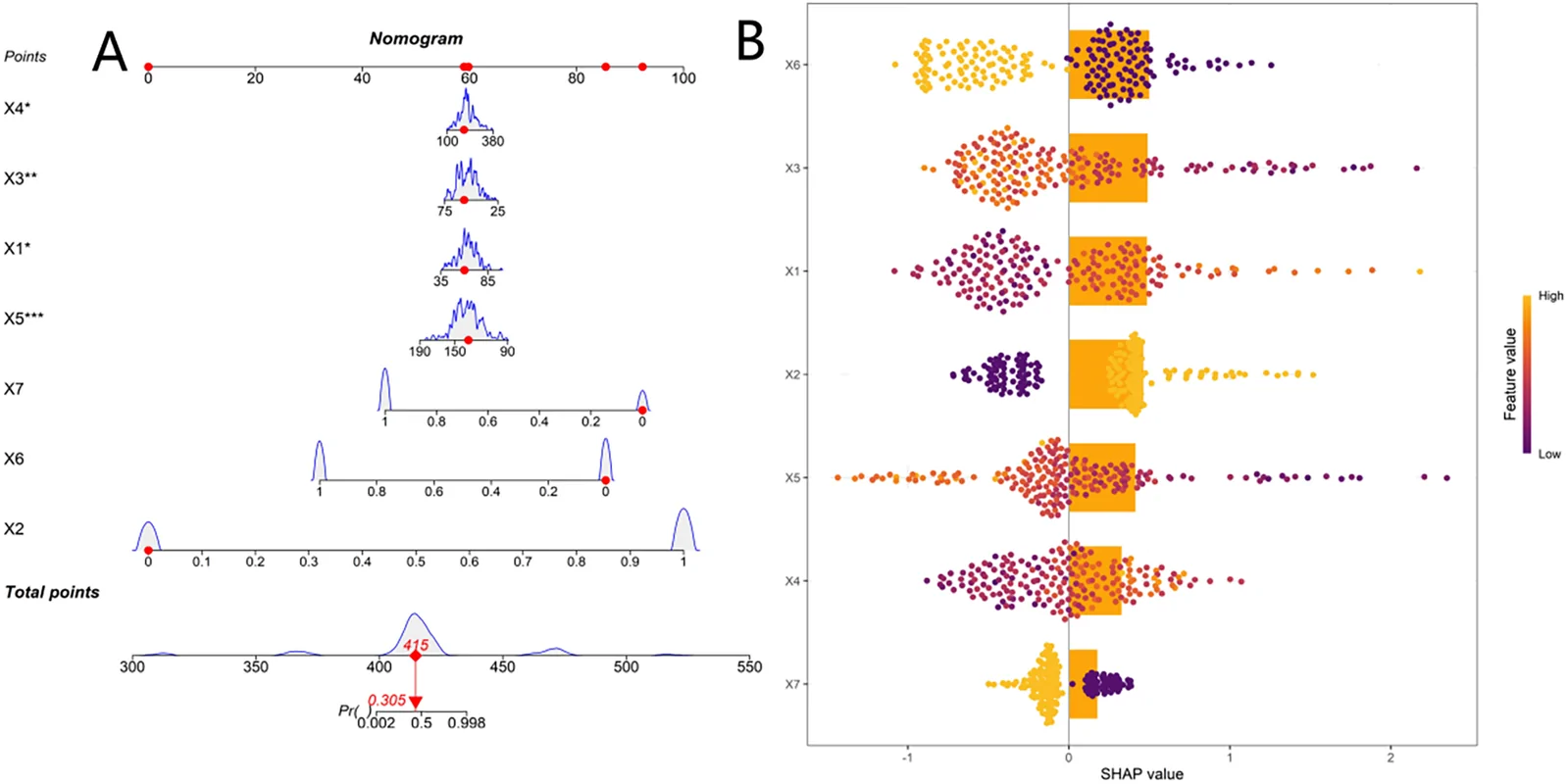
Model interpretability analysis. **(A)** Nomogram; **(B)** SHAP feature importance plot. ×1: age; ×2: type of CAD; ×3: LVEF; ×4: platelet count; ×5: hemoglobin; ×6: carriage of *CYP2C19* loss-of-function alleles; ×7: PPI use.

Based on the seven core predictive variables identified through multivariate logistic regression analysis (age, type of CAD, LVEF, platelet count, hemoglobin level, carriage of *CYP2C19* loss-of-function alleles, and PPI use), this study employed the regression model to construct a nomogram model for predicting high on-treatment platelet reactivity in patients with CAD. As illustrated in Figure 5, the nomogram intuitively displays the contribution and direction of each clinical feature towards the treatment outcome. Specifically, Figure 5A presents the weight distribution and scoring criteria for each variable in the training set, while Figure 5B provides visual validation of the model's stability and generalizability using the validation set data. The model results confirmed that age (X1), type of CAD (X2), LVEF (X3), platelet count (X4), hemoglobin (X5), carriage of *CYP2C19* loss-of-function alleles (X6), and PPI use (X7) were independent influencing factors for HPR. Increased age, acute coronary syndrome, elevated platelet count, carriage of *CYP2C19* loss-of-function alleles, and PPI use significantly increased the risk of HPR. Conversely, higher LVEF and higher hemoglobin levels emerged as protective factors, with increasing values associated with a reduced risk of HPR.

SHAP analysis further quantified the relative importance of each feature. The order of influence, from highest to lowest, was: carriage of *CYP2C19* loss-of-function alleles (X6), age (X1), platelet count (X4), hemoglobin (X5), LVEF (X3), PPI use (X7), and type of CAD (X2). Notably, carriage of *CYP2C19* loss-of-function alleles exhibited the most substantial positive predictive contribution to HPR, a finding highly consistent with its highest odds ratio (OR = 4.341) in the multivariate logistic regression. Age and elevated platelet count were also identified as significant risk factors. In contrast, LVEF and hemoglobin, as protective factors, demonstrated notable negative contributions. The SHAP analysis results were highly congruent with the conclusions from the multivariate logistic regression, confirming the central role of the pharmacogenomic marker (*CYP2C19*) in predicting antiplatelet therapy response while simultaneously clarifying the significant contributions of clinical indicators (age, platelet count, hemoglobin, LVEF, type of CAD) and drug-drug interactions (PPI use).

By integrating the nomogram with SHAP analysis, this study not only established a visualized, individualized prediction tool (as demonstrated by the training set nomogram model in Figure 5A and its validation in Figure 5B) but also elucidated the model's theoretical rationale and decision-making mechanisms from the perspective of feature importance. This significantly enhances the clinical interpretability and practical utility of the prediction results, thereby providing a theoretical foundation and practical guidance for individualized antiplatelet therapy in patients with CAD.

## 4 Discussion

CAD is a major cardiovascular disease that poses a significant threat to human health, and substantial interindividual variability exists in the response to antiplatelet therapy. Currently, effective clinical tools for predicting HPR are lacking (7). This study innovatively integrated clinical characteristics, laboratory parameters, and pharmacogenomic markers to construct and validate a logistic regression model for individualized prediction of antiplatelet therapy response in patients with CAD. The model showed promising discrimination in both the training and validation cohorts, with AUC values of 0.866 (95% CI: 0.816–0.916) and 0.870 (95% CI: 0.793–0.946), respectively. Furthermore, the robustness of the predictive variables and the stability of the model were ensured through comparisons using LASSO regression, multivariate logistic regression, and various machine learning algorithms (8–10). More importantly, an in-depth interpretability analysis of the model was conducted using a nomogram combined with SHAP analysis. This approach not only quantified the relative importance of each predictor but also elucidated their risk associations with HPR, thereby providing novel perspectives and a foundation for understanding the mechanisms underlying interindividual variability in antiplatelet therapy response and for achieving precise antithrombotic therapy in clinical practice.

Comparison with existing prediction models is instructive. The ABCD-GENE score, which includes age, BMI, chronic kidney disease, diabetes, and CYP2C19 genotype, has been validated as a simple tool to identify patients with HPR on clopidogrel. The GeneFA score, developed specifically in Chinese ACS patients, demonstrated moderate predictive ability for HPR (AUC approximately 0.78). Our model extends these approaches by incorporating additional clinically relevant variables—LVEF, platelet count, hemoglobin, and PPI use—that reflect patients' hemodynamic status, hematological profile, and potential drug-drug interactions. Furthermore, our model achieved an AUC of 0.870 in internal validation, suggesting improved discrimination compared with the GeneFA score. However, direct head-to-head comparison in the same cohort would be necessary to confirm this advantage.

The seven core predictors ultimately identified in this study profoundly reflect the nature of HPR as a complex pathophysiological process involving genetic factors, clinical characteristics, and drug interactions. Among these, carriage of a loss-of-function allele of *CYP2C19* was identified as the most influential predictor. *CYP2C19* is a critical member of the hepatic cytochrome P450 enzyme system and is involved in the metabolism of numerous drugs. Carriers of *CYP2C19* loss-of-function alleles exhibit reduced enzyme activity, leading to decreased generation of active metabolites for drugs metabolized via this pathway, consequently affecting therapeutic efficacy (11). In the present study, this factor demonstrated a high OR of 4.341 in the multivariate analysis, and the SHAP analysis also revealed its most prominent positive contribution to the model output. These findings are highly consistent with the conclusions of multiple domestic and international studies, reaffirming the central role of *CYP2C19* genetic polymorphism in the interindividual variability of antiplatelet therapy response.

Age was confirmed as the second most important risk factor, and its predictive value was validated in this model. With advancing age, declines in liver and kidney function, reduced drug-metabolizing enzyme activity, and decreased drug clearance occur. Concurrently, endothelial dysfunction and enhanced platelet activation contribute to diminished efficacy of antiplatelet agents (12). In this study, each one-year increase in age was associated with a 5.3% increase in the risk of HPR, consistent with findings from previous research. Elevated platelet count also significantly increased the risk of HPR, with a clear biological mechanism: a higher baseline platelet count necessitates greater inhibition to achieve equivalent antiplatelet effects, making patients with elevated platelet counts more susceptible to inadequate inhibition under standard-dose antiplatelet therapy.

Notably, hemoglobin level and LVEF were identified as significant protective factors (13). Reduced hemoglobin levels often indicate anemia. While the negative association between anemia and HPR is not yet fully understood, it may be related to compensatory hemodynamic alterations or inflammatory states associated with anemia. Reduced LVEF reflects impaired cardiac systolic function. Patients with this condition often exhibit neuroendocrine system activation, increased sympathetic activity, and release of inflammatory cytokines, which may indirectly affect platelet activity and drug response. The identification of these two protective factors provides new insights for clinical assessment (14).

Type of CAD and use of PPIs, as categorical variables, also demonstrated significant predictive value (15). Patients with ACS are in a hypercoagulable state with high platelet activation and strong inflammatory responses, making them more susceptible to HPR compared to those with stable CAD. PPIs, particularly omeprazole and esomeprazole, interact with some drugs metabolized by *CYP2C19* by competitively inhibiting *CYP2C19* enzyme activity, thereby affecting drug metabolism. In this study, PPI users had a 2.63-fold higher risk of HPR compared to non-users, suggesting that careful selection of PPIs is crucial in clinical practice when co-administering drugs metabolized by *CYP2C19*, with preference given to agents such as pantoprazole that have less competitive inhibition of *CYP2C19*.In our study, *ABCB1* and *PON1* polymorphisms did not show statistically significant associations with HPR in univariate analysis and were not retained in the final model. This finding is consistent with several recent studies that have reported inconsistent or negative associations between these variants and clopidogrel response. The lack of association may be due to the relatively small sample size for detecting modest genetic effects or population-specific allele frequencies.

The core innovation of this study lies in its transcendence of traditional predictive approaches that primarily rely on clinical characteristics, achieving a multidimensional integration of clinical indicators, laboratory tests, and pharmacogenomic markers (16). Our predictive model demonstrates that the response to antiplatelet therapy is not determined by a single factor but is instead a product of genetic background (*CYP2C19* gene), underlying disease status (type of CAD, cardiac function), hematological status (platelet count, hemoglobin level), and drug interactions (PPI use) (17, 18). This finding strongly underscores the important value of precision medicine principles in the management of cardiovascular diseases.

Methodologically, this study combined the strengths of traditional statistical methods with modern machine learning techniques (19). Feature selection was performed using LASSO regression to avoid model overfitting. Subsequently, multivariate logistic regression was employed to determine the independent effects and risk ratios of each factor. Finally, a logistic regression algorithm was utilized to construct a model with superior predictive performance and linear interpretability, while random forest was used for comparison. This combinatorial strategy ensured both the statistical rigor of the model and enhanced predictive accuracy. Furthermore, the application of SHAP interpretability analysis represents a significant highlight of this study. It objectively quantified the contribution ranking of each feature and clearly illustrated the direction (positive or negative) of each feature's contribution to individual predictions. This “white-box” approach greatly enhances clinicians' trust and understanding of the model's decision-making process, facilitating a shift in predictive models from a focus solely on “predictive performance” toward “clinical trustworthiness.”.

Several limitations of this study should be acknowledged. First, this was a single-center, retrospective study. Although internal validation was performed, the generalizability of the model requires further validation in multicenter, prospective external cohorts. Second, all predictors were derived from baseline assessments, and dynamic changes during treatment or data on drug concentrations were not incorporated. Third, not all genetic loci potentially influencing antiplatelet response were included. While genes such as *ABCB1* and PON1 showed no statistical significance in the univariate analysis of this study, their potential roles warrant further exploration in larger sample-sized studies (20–22). Fourth, all patients in this study were of Han Chinese ethnicity. Given that Qinghai Province is home to multiple ethnic groups and *CYP2C19* allele frequencies vary by ancestry, the generalizability of our findings to other ethnic populations requires further validation. Fifth,although we collected detailed information on PPI type, the sample size was insufficient for subtype-specific stratified analyses. Given that different PPIs vary substantially in their CYP2C19 inhibitory effects, future large-scale studies are needed to evaluate the differential impact of individual PPIs on clopidogrel response. Additionally, data on PPI dose, duration, and precise temporal overlap were not available due to the retrospective design. Finally, this study focused on a single antiplatelet agent. Future research could be extended to other types of antiplatelet drugs to construct more universally applicable predictive models.

In summary, this study constructed and validated a predictive model for antiplatelet therapy response in CAD that integrates clinical characteristics, laboratory parameters, and pharmacogenomic markers. This model not only exhibits stronger predictive performance but also, through interpretability analysis, provides in-depth insights into the key factors influencing HPR and their underlying logic. The findings emphasize the central value of *CYP2C19* genetic testing in guiding individualized antiplatelet therapy, while also clarifying the significant roles of age, CAD type, cardiac function, platelet count, hemoglobin level, and PPI use. While this model shows promise as a potential clinical decision-support tool for identifying patients at higher risk of HPR, prospective evaluation in external cohorts is essential before it can be considered for clinical implementation. Future efforts should focus on multicenter prospective external validation and explore the incorporation of treatment process parameters and novel antiplatelet agents into the model to further enhance its clinical utility.

## Data Availability

The raw data supporting the conclusions of this article will be made available by the authors, without undue reservation.
