# Supplementary material for: Development and validation of a clinical-pharmacogenomic prediction model for clopidogrel-related high on-treatment platelet reactivity in patients with coronary artery disease
Source: Front Cardiovasc Med. 2026 Sep 3;13:1926514. doi: 10.3389/fcvm.2026.1926514 (PMC13584308; doi:10.3389/fcvm.2026.1926514)
Supplement: Supplementary file 1 [file Table1.docx]

| **Variant** | **Genotype** | **n (%)** | **Allele** | **Frequency (%)** | **HWE P-value** |
| --- | --- | --- | --- | --- | --- |
| *CYP2C19* *2 (rs4244285) | GG | 196 (51.6) | G | 71.8 | 0.324 |
|  | GA | 153 (40.3) | A | 28.2 |  |
|  | AA | 31 (8.2) |  |  |  |
| *CYP2C19* *3 (rs4986893) | GG | 352 (92.6) | G | 96.3 | 0.587 |
|  | GA | 28 (7.4) | A | 3.7 |  |
|  | AA | 0 (0.0) |  |  |  |
| *CYP2C19* *17 (rs12248560) | CC | 316 (83.2) | C | 91.2 | 0.456 |
|  | CT | 61 (16.0) | T | 8.8 |  |
|  | TT | 3 (0.8) |  |  |  |
| *ABCB1* rs1045642 | CC | 94 (24.7) | C | 50.0 | 0.892 |
|  | CT | 191 (50.3) | T | 50.0 |  |
|  | TT | 95 (25.0) |  |  |  |
| *PON1* rs662 | QQ | 186 (48.9) | Q | 69.7 | 0.753 |
|  | QR | 160 (42.1) | R | 30.3 |  |
|  | RR | 34 (8.9) |  |  |  |

**Supplementary Table 1**. Genotype and allele frequencies of the genotyped variants
