# Supplementary material for: Development and validation of a clinical-pharmacogenomic prediction model for clopidogrel-related high on-treatment platelet reactivity in patients with coronary artery disease
Source: Front Cardiovasc Med. 2026 Sep 3;13:1926514. doi: 10.3389/fcvm.2026.1926514 (PMC13584308; doi:10.3389/fcvm.2026.1926514)
Supplement: Supplementary file 2 [file Table2.docx]

**Supplementary Table 2.** Variable assignment

| Variable | Meaning | Assignment |
| --- | --- | --- |
| X1 | Age | Continuous variable |
| X2 | Type of CAD | Stable coronary heart disease = 0, Acute coronary  syndrome = 1 |
| X3 | LVEF | Continuous variable |
| X4 | Platelet count | Continuous variable |
| X5 | Hemoglobin | Continuous variable |
| X6 | Carriage of *CYP2C19* loss-of-function alleles | Non-carrier = 0, Carrier = 1 |
| X7 | PPI use | Yes = 1, No = 0 |
| Y | Final diagnosis | Normal platelet reactivity group = 0, High on-treatment platelet reactivity group = 1 |
